# Automatic Multichannel Electrocardiogram Record Classification Using XGBoost Fusion Model

**DOI:** 10.3389/fphys.2022.840011

**Published:** 2022-04-14

**Authors:** Xiaohong Ye, Yuanqi Huang, Qiang Lu

**Affiliations:** ^1^ Chengyi University College, Jimei University, Xiamen, China; ^2^ School of Physical Education and Sport Science, Fujian Normal University, Fuzhou, China; ^3^ School of Science, Jimei University, Xiamen, China

**Keywords:** electrocardiogram (ECG), classification algorithm, physiological signal processing, bioengineering, model fusion, extreme gradient boosting (xgboost), 12-lead

## Abstract

There is an increasing demand for automatic classification of standard 12-lead electrocardiogram signals in the medical field. Considering that different channels and temporal segments of a feature map extracted from the 12-lead electrocardiogram record contribute differently to cardiac arrhythmia detection, and to the classification performance, we propose a 12-lead electrocardiogram signal automatic classification model based on model fusion (CBi-DF-XGBoost) to focus on representative features along both the spatial and temporal axes. The algorithm extracts local features through a convolutional neural network and then extracts temporal features through bi-directional long short-term memory. Finally, eXtreme Gradient Boosting (XGBoost) is used to fuse the 12-lead models and domain-specific features to obtain the classification results. The 5-fold cross-validation results show that in classifying nine categories of electrocardiogram signals, the macro-average accuracy of the fusion model is 0.968, the macro-average recall rate is 0.814, the macro-average precision is 0.857, the macro-average F1 score is 0.825, and the micro-average area under the curve is 0.919. Similar experiments with some common network structures and other advanced electrocardiogram classification algorithms show that the proposed model performs favourably against other counterparts in F1 score. We also conducted ablation studies to verify the effect of the complementary information from the 12 leads and the auxiliary information of domain-specific features on the classification performance of the model. We demonstrated the feasibility and effectiveness of the XGBoost-based fusion model to classify 12-lead electrocardiogram records into nine common heart rhythms. These findings may have clinical importance for the early diagnosis of arrhythmia and incite further research. In addition, the proposed multichannel feature fusion algorithm can be applied to other similar physiological signal analyses and processing.

## 1 Introduction

Cardiovascular diseases (CVDs) are the most common cause of death, accounting for more than 31% of the world’s deaths (Roth et al., 2018). Among these diseases, more than 80% of sudden cardiac deaths are closely related to cardiac arrhythmia (CA) (Anderson et al., 2010), accounting for half of all cardiac deaths. Moreover, many types of CA are life-threatening and are possibly caused by various cardiac diseases, such as cardiomyopathy, myocardial infarction and myocarditiss (Afonso and Tompkins, 1995; Minami et al., 1999). Therefore, automatically identifying and classifying electrocardiogram (ECG) (Joshi et al., 2014) data using computers can improve the efficiency and accuracy of CA diagnosis and relieve doctors from the tedious work of pattern recognition. It is the foundation of machine-aided diagnosis and treatment of cardiovascular diseases and lays an important foundation for the future development of wearable devices.

The traditional classification method of ECG signals based on feature extraction requires human experts to engineer useful features based on raw ECG data, which are referred to as “expert features”. Then, deployed decision rules or other machine learning (ML) methods generate the final results. Expert features can be categorized into statistical features [such as sample entropy (Alcaraz et al., 2010), heart rate variability (HRV) (Malik et al., 1996), and coefficients of variation and density histograms (Tateno and Glass, 2001)], frequency-domain features (Lin, 2008) and time-domain features. However, such traditional methods have reached their limit on performance because they are restricted by data quality and human expert knowledge (Schläpfer and Wellens, 2017). Recently, deep neural networks (DNNs) have proven their potential for different classification tasks (Ker et al., 2018; Ker et al., 2019a; Ker et al., 2019b; Yadav and Jadhav, 2019). In contrast from traditional methods, DNNs can learn a feature extraction function from the raw input based on the probability distribution of the dataset. They have also been applied to ECG processing and have achieved an outstanding performance (Baloglu et al., 2019; Hannun et al., 2019).

A recent study (Hannun et al., 2019)showed that for single-lead ECGs, DNNs could match state-of-the-art algorithms when trained on openly available datasets, and for a sufficiently large training dataset, DNNs present superior performance when compared to practising cardiologists. However, as Hannun et al. pointed out, it is still an open question whether the application of this technology would be useful in a realistic clinical setting. In a realistic clinical setting, 12-lead ECGs (S12L-ECGs) are the standard, where a complete ECG usually contains a 12-lead signal from six limb leads (I, II, III, aVR, aVL, aVF) and six chest leads (V1, V2, V3, V4, V5, V6) (Wilson et al., 1954). The different leads exhibit distinct features of ECG signals that are associated with specific types of CA (Ashley and Niebauer, 2004; Lacalzada-Almeida et al., 2020). Therefore, the automatic calculation and analysis of the standard S12L-ECG is becoming increasingly important for medical diagnosis.

Moreover, different network frameworks illustrate different performances when using deep neural networks to classify S12L-ECG signals. The results of these works show that combining convolutional neural network (CNN) (Fukushima and Miyake, 1982) and recurrent neural network (RNN) (Goller and Küchler, 1996) modules is the preferred architecture for handling ECG signals with varied sequence lengths and multichannel inputs (Liu et al., 2019; Yildirim et al., 2019; Li et al., 2020; Shaker et al., 2020). This may be because CNN is an effective method for extracting features due to its local connectivity and parameter sharing, and RNN is used for processing time-series signals since the ECG signal records the time course of cardiac electrical activities. For example, (Yao et al., 2018) proposed a time-incremental CNN (TI-CNN) integrating a VGGNet-based CNN and long short-term memory (LSTM) layers for CA classification, which utilised recurrent cells to introduce flexibility in input length for CNN models. This was important to solve the problem that the varied-length signal could not be accepted by CNN models. (He et al., 2019) proposed a model consisting of a deep residual network (ResNet) and a bi-directional LSTM (BiLSTM) layer, which obtained a good performance in classifying 9 CA classes without filtering. Although these architectures based on combining CNN and RNN modules make good use of the modules to extract local and temporal features and achieve a reasonable performance, the following points are ignored in the architectures. First, periodicity exists in ECG signals, meaning that localised waveform features and statistical features both contribute to CA detection and should therefore be emphasized (Tseng et al., 2016). Second, ECG signals of different patients under different physical conditions have distinctive morphological and temporal features (Banerjee and Mitra, 2014). Therefore, different patients with the same disease may have diverse ECG morphologies. (Dilmac and Korürek, 2015; Mateo et al., 2016; Shadmand and Mashoufi, 2016). Third, widely applied S12L-ECG recordings provide richer information for diagnosing CAs. The different leads of S12L-ECG exhibit distinct features of ECG signals that are associated with specific types of CA, so they have different contributions for detecting CA. For example, left bundle branch block (LBBB) is diagnosed by distinct QRS morphology at leads I, aVL, V1, V2, V5, and V6, while right bundle branch block (RBBB) is diagnosed by the rsR’ pattern at V1 and V2 (Dale, 2000; Surawicz et al., 2009; Ikeda, 2021); The patient with STD has an S-peak in leads I, V5, V6, and aVL that are 3 mm lower than the PR segment, and may have normal rhythms in leads V2-V3 (Fred, 2020). Therefore, effectively fusing information from 12 leads, along with troubles it might bring, should be considered in the model design (Jordaens, 2018).

Therefore, we consider the above points and combine the feature-based method to customize the model according to the characteristics of 12-lead ECG recording. Specifically, considering the first point, we add HRV statistical features in our method. Considering the second point, we add age and sex, which can reflect more specific patient information for the features. Considering the third point, we use multiple CNNs to extract the local features of the pre-processed signal segments, and then use BiLSTM to enhance the temporal features between ECG signal segments to obtain a single-lead CNN-BiLSTM network. Finally, 12 CNN-BiLSTMs and domain-specific features (DFs: HRV, age, and sex features) are fused based on eXtreme Gradient Boosting (XGBoost) to make full use of the information from the S12L-ECG records. The proposed method classifies S12L-ECG records into nine categories, including normal signals and eight abnormal signals of four categories (Li et al., 2018), and demonstrated a high performance in classifying S12L-ECG signals.

## 2 Materials and Methods

**FIGURE 1:**
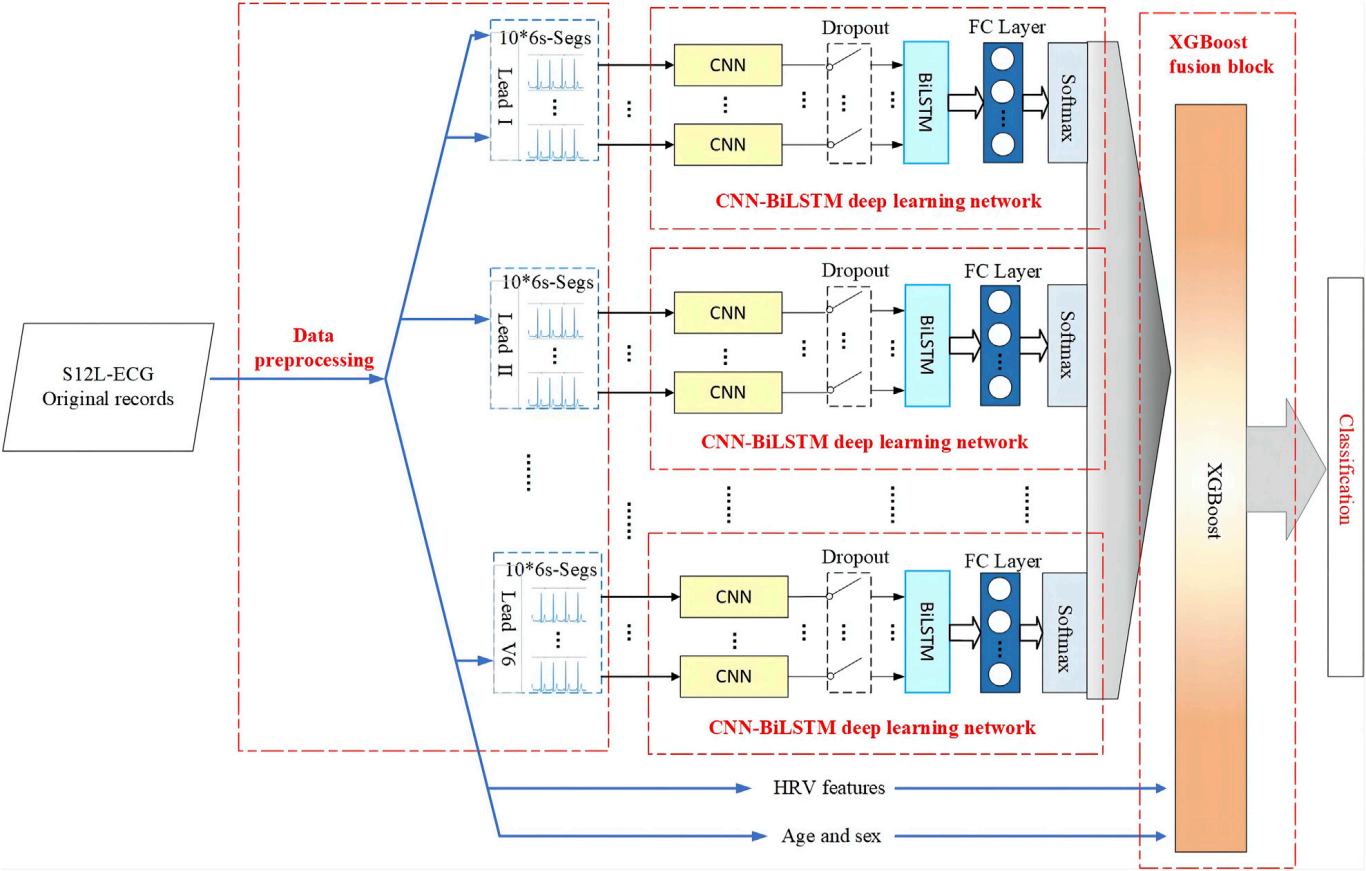
Flowchart diagram of the proposed method for S12L-ECG record classification.

The flowchart diagram of the proposed S12L-ECG record classification method is shown in Figure 1, which includes four parts: data pre-processing, the CNN-BiLSTM deep learning network, the XGBoost fusion block, and classification.

### 2.1 Materials

**FIGURE 2:**
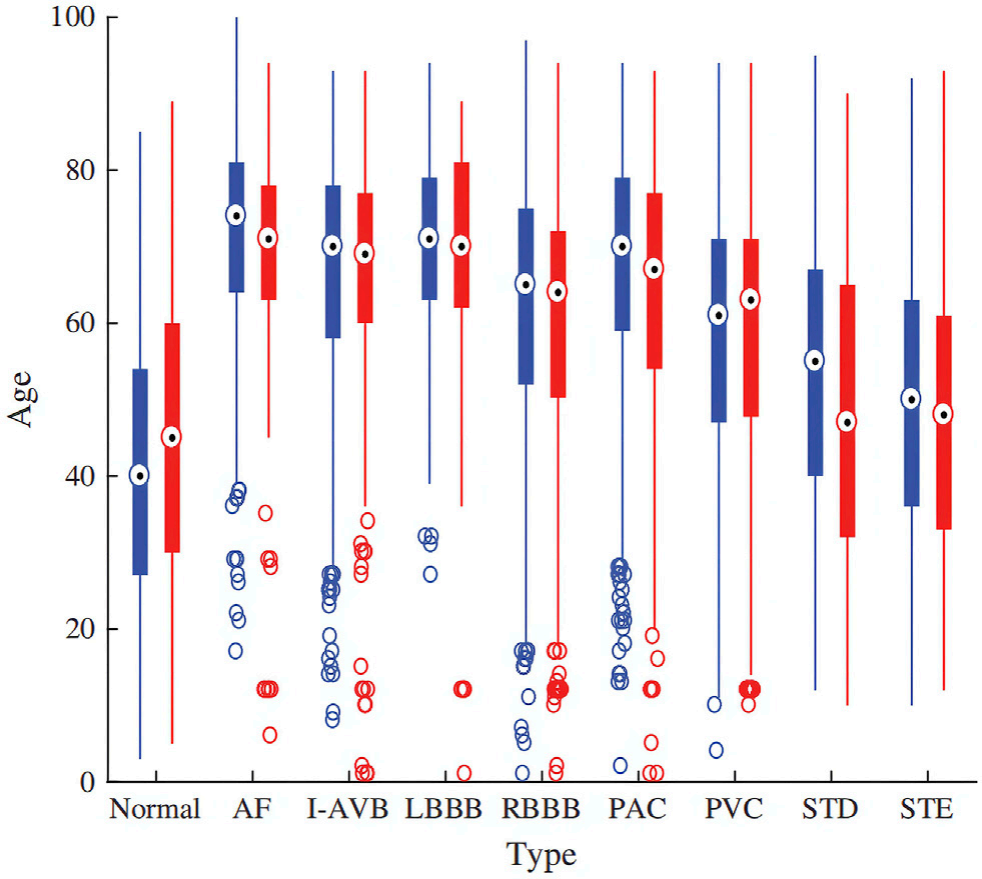
The age distribution of the dataset.

In this study, we use the dataset of the 2018 China Physiological Signal Challenge (CPSC 2018) (Liu et al., 2018), which is collected from 11 hospitals. The training set of dataset contains a total of 6877 S12L-ECG records for 3,699 males and 3,178 females. In addition, there are 2,954 records as a test set which is not public. Signals were sampled at 500 Hz, and each record contained a standard S12L-ECG signal, along with the age and sex of the individual. Figure 2 illustrates the patients’ age distribution. Each signal has an uncertain length ranging from 6 to 60 s. In this dataset, the ECG records contain normal heart rhythm and eight types of CA. Most records have only one label, but some records have two or three labels because the patient providing the signals has multiple diseases at the same time. There are 477 and 203 subjects of this multi-label type in the training and test sets, respectively. The details of the dataset used are described in Table 1. For the records with multiple labels, the statistics in Table 1 are based on the first label.

**TALBE 1:** Data profile for the dataset.

Dataset	Type	Number of records	Time length (s)				
			Min	Median	Max	Mean	SD
**Training Set**	Normal (**N**)	918	10.00	13.00	60.00	15.43	7.61
	Atrial fibrillation (**AF**)	1,098	9.00	11.00	60.00	15.01	8.39
	First-degree atrioventricular block (**I-AVB**)	704	10.00	11.27	60.00	14.32	7.21
	Left bundle branch block (**LBBB**)	207	9.00	12.00	60.00	14.92	8.09
	Right bundle branch block (**RBBB**)	1,695	10.00	11.19	60.00	14.42	7.60
	Premature atrial contraction (**PAC**)	556	9.00	14.00	60.00	19.46	12.36
	Premature ventricular contraction (**PVC**)	672	6.00	15.00	60.00	20.21	12.85
	ST-segment depression (**STD**)	825	8.00	12.78	60.00	15.13	6.82
	ST-segment elevated (**STE**)	202	10.00	11.89	60.00	17.15	10.72
	**Total/Average**	6,877	6.00	12.00	60.00	15.79	9.04
**Test Set**	Normal (**N**)	394	—	—	—	15.91	—
	Atrial fibrillation (**AF**)	469	—	—	—	17.31	—
	First-degree atrioventricular block (**I-AVB**)	301	—	—	—	15.34	—
	Left bundle branch block (**LBBB**)	90	—	—	—	16.51	—
	Right bundle branch block (**RBBB**)	729	—	—	—	16.53	—
	Premature atrial contraction (**PAC**)	250	—	—	—	23.06	—
	Premature ventricular contraction (**PVC**)	281	—	—	—	21.28	—
	ST-segment depression (**STD**)	354	—	—	—	14.93	—
	ST-segment elevated (**STE**)	86	—	—	—	22.46	—
	**Total/Average**	2,954	—	—	—	18.15	—

The mark “—” is filled when the data was not given.

### 2.2 Pre-Processing

We pre-process the data before inputting it into the model, including information separation, downsampling, oversampling, filtering, signal slicing, and the Z-Score.

#### 2.2.1 Information Separation

In the dataset, each record is a file in mat format. In addition to the S12L-ECG signal data, the file also contains age and sex information. We extract age and sex information from the record files and save them as a csv format file.

#### 2.2.2 Downsampling

The sampling rate of the ECG signal in the dataset is 500 Hz. To reduce the computational burden, we downsample all signals to 250 Hz. The downsampling operation speeds up the training process and has almost no loss of information from the ECG signals.

#### 2.2.3 Oversampling

From the distribution of record types in Table 1, we can see the sample imbalance in the dataset, which has a negative impact on training the model. Therefore, we use an oversampling method to balance different types of data in the dataset. Specifically, we randomly divides five subsets for each class, and take the category with the largest number of samples as a reference. When the sample size of other categories is only half or less of the reference category, we will directly carry out the corresponding multiple replication; otherwise, we will randomly select samples in this category to replicate the missing quantity. This solves the problem of imbalance, and also processes five subsets for 5-fold cross-validation.

#### 2.2.4 Filtering

The ECG signal is a weak physiological electrical signal that is easily disturbed by noise. Wavelet theory has already proven its ability to split signals and noise in the wavelet domain. Researchers from the biomedical signal processing community have applied wavelet theory in denoising and have shown a superior performance (Alyasseri et al., 2018). The DBN wavelet basis functions constructed by Daubechies have good symmetry and regularity and do not easily produce phase distortion, which makes the reconstructed signal smoother. Among them, the DB6 wavelet basis function is closer to the ECG signal and has a better effect on denoising the ECG signal (Saxena and Vijay, 2020). Therefore, we subjected the ECG signal to wavelet-based denoising using the DB6 wavelet basis function. Specifically, we use the DB6 wavelet to decompose the ECG signal up to nine levels. The ninth level approximation sub-band (the low frequency component A9) contains a frequency range of 0–0.351 Hz, which is mainly the baseline wander, and is not used for reconstructing the denoised signal. Additionally, the ECG would not contain much information after 45 Hz. Therefore, the first- and second-level detail coefficients (the high-frequency components D1 and D2) consisting of frequency bands of 90–180 Hz and 4–90 Hz, respectively, are not used for reconstructing the denoised ECG. The required sub-bands, the 3rd-, 4th-, 5th-, 6th-, 7th-, 8th- and 9th-level detail signals are only used (all other sub-band coefficients were replaced with zeros) to compute the inverse wavelet transform to obtain the filtered ECG signal (Martis et al., 2013).

#### 2.2.5 Signal Slicing

The length of the record sequence in the dataset is not fixed with many long sequences. On the one hand, the longer the length of the record, the greater the amount of information and corresponding features. However, if the entire record is input, to effectively extract the local features of the ECG signal, it is necessary to configure more convolution kernels in each layer, which causes the entire network to become very bloated. On the other hand, the length of the record sequence in the dataset is not fixed, and CNN generally accepts inputs of the same length. Once the sequence length has a relatively large variation range, it needs to be made equal through large artificial extension or up and down sampling, which will have a certain destructive effect on the data information. Therefore, it is not appropriate to directly use the entire record as input. To solve this problem, the record can be segmented according to heartbeats or divided into short segments of a certain length. Although the results of the heartbeat-based algorithm in the ECG signal classification work are fine enough to be implemented in a single heartbeat, additional QRS wave detection algorithms or manual positioning of the heartbeat are required. Thus, the back-end classifier depends on the specific positioning method. In addition, our network architecture design requires the same length and the same number of segment inputs, maintaining the temporal characteristics between segments. Since the lengths of the ECG sequences in the dataset are unequal, if the heartbeats are extracted, the number of heartbeats in each ECG sequence cannot be the same while maintaining the time characteristics between the heartbeats.

**FIGURE 3:**
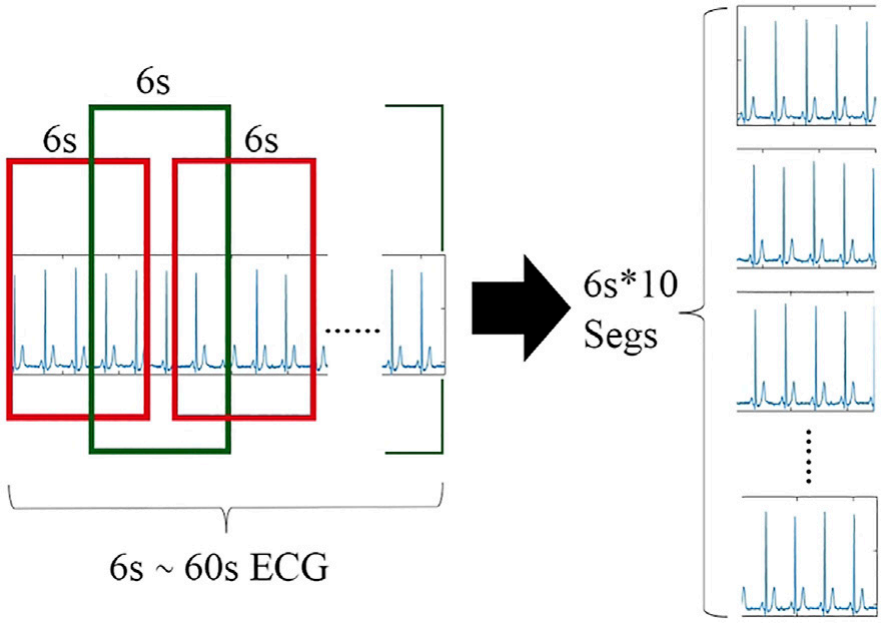
Slicing process of the single lead ECG signal sequence.

Therefore, we propose an algorithm to extract 10 segments (6 s long) from each record sequence in turn and then stack them again. We determine the category label of the parent ECG as the category of the child. For records with multiple labels, we directly take the first category label of the parent ECG as the category of the child. In addition, we also apply other segment lengths (such as 3 and 10 s); however, it proves that 6 s is the optimal choice. As shown in Table 1, the record length ranges from 6 to 60 s. Thus, when a segment is 6 s, if the record length is the shortest at 6 s, it can be copied directly 10 times, minimizing the artificial expansion. The longest 60 s can cut 10 segments without overlap. Only the sequence in the middle length will have varying degrees of overlap between the slice fragments. In this way, the data in the dataset keep the original information and the least redundancy as much as possible. After processing, each lead of each record sequence is a set of 6s × 10 sequence segments, which still has time features. The slicing process of the single-lead ECG signal sequence is shown in Figure 3.

#### 2.2.6 Z-Score

For a large average and a wide range of data, it is usually necessary to standardize the data before entering the network. We use the Z-Score, which is a commonly used data standardization method, to standardize the slice fragments. The mean value of the processed data is 0, and the standard deviation is 1. The conversion formula is shown in Eq. 1.

$$x^{*}=\frac{x-\bar{x}}{\sigma }$$ (1)

where x is the original data, $\bar{x}$ is the mean value of the original data, and σ is the standard deviation of the original data (Kamphaus, 2000).

### 2.3 Model Architecture

The proposed model illustrated in Figure 1 is composed of multiple fully CNN subnetworks, 12 BiLSTM networks and an XGBoost network. For a set of 6s × 10 sliced segments in each lead, we designed a simple 1-dimensional (1-D) fully connected CNN to extract the local features of the ECG sliced segment. Each lead of the ECG signal corresponds to 10 CNN subnetworks. For each ECG lead signal, the output of the CNN subnetworks is spliced in the order of the signal segment as the input of the BiLSTM network. Following the CNN subnetworks are 12 BiLSTM networks that are used to enhance the temporal information between ECG signal segments according to the features of the ECG sequence. Finally, XGBoost is used to fuse 12 CNN-BiLSTM deep learning networks and corresponding DFs for each ECG record. Then, we output the final classification results.

#### 2.3.1 CNN Subnetwork

**FIGURE 4:**
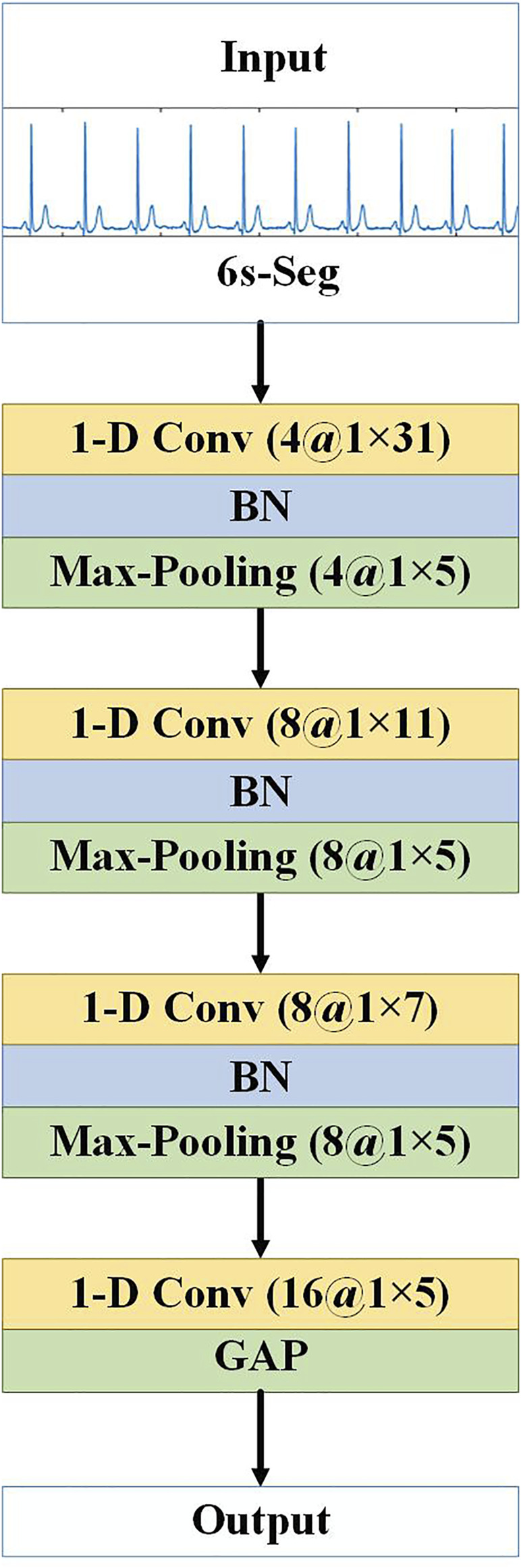
Architecture of CNN subnetwork. “1-D Conv” represents the 1-D convolutional layer, before “at” is the number of convolution kernels, and after “at” is the convolution kernel size. “Max-Pooling” is the maximum pooling layer, before “at” is the number of feature maps, and after “at” is the pooling step size.

We use CNNs to extract the local features of the ECG signal segments. The 10 segments of a lead are input into different branches as a whole, and each branch is a 1-D CNN network that processes a 6 s ECG signal segment. The network architecture is shown in Figure 4. There are four 1-D convolutional (1-D Conv) layers and three max-pooling layers, which alternately extract abstract local waveform features in an ECG signal slice segment. Each convolutional layer is followed by a batch normalization (BN) layer (Ioffe and Szegedy, 2015) to accelerate the convergence of the network, prevent gradient diffusion, and to a certain extent, prevent the influence of overfitting. Use the rectified linear unit (ReLU) as activation function. Finally, the global average pooling (GAP) layer replaces the fully connected layer of the traditional CNN. The GAP layer pools each feature map of the last convolution to obtain a mean. Pooling operations do not require parameter updates; thus, the number of parameters can be reduced, and the network training time can be shortened. In addition, the GAP layer can in turn act as a regularisation to prevent overfitting, and the feature semantics extracted from the convolution and max-pooling layers are retained, which substantially improves the effect in practical applications. Nevertheless, it should be noted that the proposed model was designed to be compatible with a variety of CNN designs, and in other signal processing problems, the depth and design of convolutional layers could be adjusted based on the specific requirements of those problems.

#### 2.3.2 The CNN-BiLSTM Deep Learning Network

**FIGURE 5:**
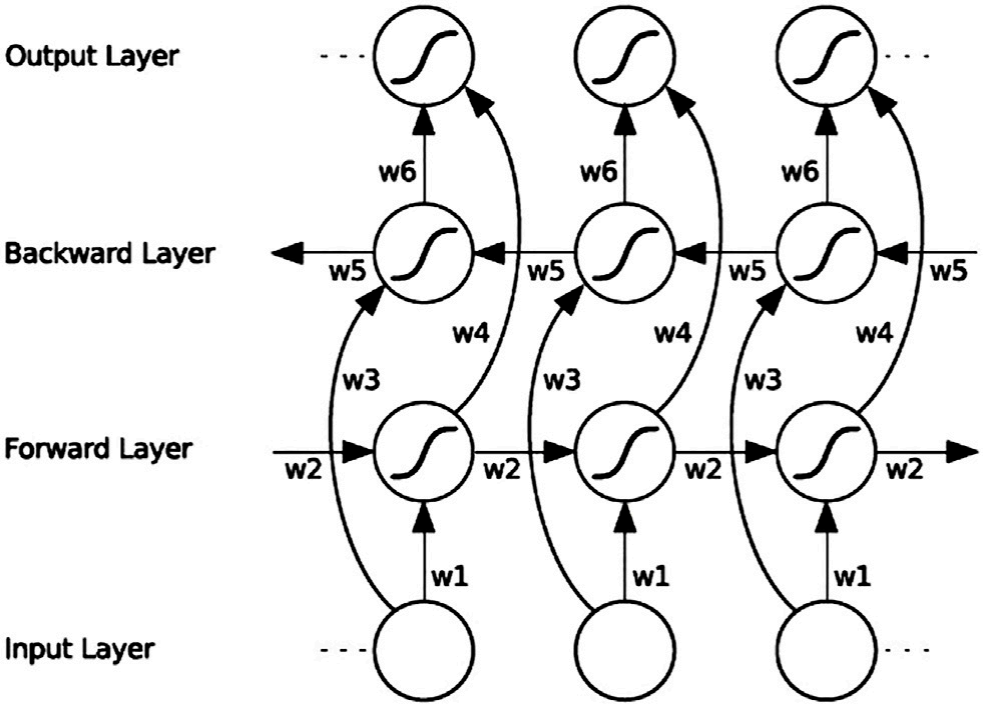
BiLSTM architecture diagram. “w1” ~ “w6” are the six special weights that are reused at each time step, corresponding respectively: input to the forward and backward hidden layers (w1, w3), and from the hidden layer to the hidden layer (w2, w5), forward and backward hidden layer to output layer (w4, w6).

RNN is a type of neural network used for processing time-series signals, and the output of its neurons will act on the output of the next time period. Since the ECG signal records the time course of cardiac electrical activities, RNN is also applied for ECG signal classification. However, RNNs cannot solve the problem of long-term dependence, and for very long sequences, RNNs will experience gradient disappearance and gradient explosion. The LSTM (Hochreiter and Schmidhuber, 1997) proposed for this problem can learn long-term dependencies. LSTM is a typical variant of RNN. Based on RNN, LSTM adds three logic control gate units: an input gate, a forget gate and an output gate. By controlling the forget gate, input gate, and output gate calculated according to the current input and the hidden state at the previous moment, important information is retained and unimportant information is forgotten, thereby eliminating the gradient dispersion problem that exists in RNN. Although LSTM solves the long-term dependency problem, it cannot encode back-to-front information. BiLSTM can evaluate the bi-directional information of the sequence in the time range, which is a type of LSTM. In many fields, such as speech recognition (Graves et al., 2013), natural language processing (Mikolov et al., 2012), and sequence classification (Ren et al., 2017), BiLSTM performance has surpassed LSTM. BiLSTM is a combination of forward LSTM and backward LSTM. A BiLSTM network expanded over time is shown in Figure 5.

BiLSTM uses the forward LSTM to obtain the above information of the input sequence, uses the backward LSTM to obtain the following information of the input sequence, and then calculates the final hidden state through vector splicing, as in:

$$h_{i}=\vec{h_{t}}⊕\overset{\leftarrow }{h_{t}}$$ (2)

where $\vec{h_{t}}$ is the hidden state of the positive LSTM output at time t. $\overset{\leftarrow }{h_{t}}$ is the hidden state of the reverse LSTM output (Bin et al., 2019).

For the signal of each ECG lead, the 10 CNN subnetwork outputs corresponding to the 10 slice segments are spliced according to the time sequence of the signal segments and used as the input of a BiLSTM network to enhance the spatiotemporal information between the segments. Because most of the segments overlap, to reduce information redundancy to prevent overfitting, this paper adopts the dropout strategy (keep_prob is set to 0.5) between CNNs and BiLSTM; thus, some neurons are randomly deleted in each iteration, which achieves the effect of regularisation to a certain extent and improve generalisability. Finally, the fully connected layer (FC layer) is connected to a vector and sent to softmax to obtain the probability value of each category. The single-lead CNN-BiLSTM deep learning network is shown in Figure 1. Each ECG lead corresponds to a CNN-BiLSTM deep learning network. Thus, each S12-ECG signal corresponds to 12 CNN-BiLSTM deep learning networks.

#### 2.3.3 The Fusion Model Using Extreme Gradient Boostingt

XGBoost (Chen and Guestrin, 2016) is improved based on the gradient boosted decision tree (GBDT) and is a parallel regression tree model that combines the idea of boosting, integrating weak classifiers into a strong classifier. Compared with the GBDT model, XGBoost overcomes the limited calculation accuracy and speed. The XGBoost algorithm serialises multiple regression trees. Besides the first regression tree, each regression number predicts the residual error; therefore, the final predicted value of the XGBoost model is the sum of the values of each regression tree. As the number of iterations increases, the accuracy continues to improve. In addition, regularisation is performed when constructing the regression tree, allowing column sampling to prevent overfitting. In practice, the XGBoost algorithm has shown good results in many prediction fields (Wang and Guo, 2019).

HRV is the change in the difference in heartbeat cycles from time to time (Uhlig et al., 2020). HRV contains information on the regulation of the cardiovascular system by neurohumoural factors. It can be used to evaluate the condition of cardiovascular diseases and is an important index for the clinical prediction of sudden cardiac death or other arrhythmic ailments. Some articles use specific waveform parameters, such as the QRS interval, PR interval, and ST segment slope, which are directly linked to medical experience, as features to determine the ECG signal category. However, in addition to the relatively mature QRS main wave positioning algorithm, other reference points, such as the P wave and its starting and ending points and the T wave starting and ending points, are more difficult to accurately locate, and their robustness is far less than that of QRS wave positioning. Therefore, it is a robust choice for HRV analysis based on QRS wave positioning to obtain the RR interval. At the same time, HRV analysis is often used for diagnosing abnormalities, such as atrial fibrillation and premature beats and has corresponding medical importance. Nevertheless, we use a CNN to automatically learn features. The strength of the CNN lies in the extraction of local detailed features, while the features in HRV analysis, such as calculating the standard deviation of the RR interval, are a typical statistical feature. Additionally, there is an abundance of literature that asserts the role of age and sex in the development of cardiovascular diseases (Shih et al., 2019). Therefore, in addition to the features extracted by our previous CNN-BiLSTM deep learning network, adding some HRV features as well as age and sex as auxiliary features, will help improve the classification performance of the algorithm.

After pre-processing the ECG records, because all of the ECG leads are collected at the same time and are completely parallel in time, we use the GQRS algorithm provided by PhysioNet, a commonly used QRS detection algorithm, to generate the HRV signal from lead II. Based on the HRV signal, we computed the following different features:

1) Standard deviation of RR intervals (SDRR).
2) Longest RR interval.
3) Shortest RR interval.
4) Mean RR interval (meanRR).
5) NN50 count divided by the total number of all RR intervals (pNN50).
6) Root mean square of differences between the adjacent RR intervals (RMSSD).
7) Sample Entropy of RR interval (SampEn).

We take each ECG lead signal in the record as a branch and input it into the CNN-BiLSTM deep learning model to obtain the softmax probabilities of nine categories. Considering that the probabilities of all categories add up to 1, to remove redundant information, we take the first eight probabilities. Since 12 leads of each ECG signal have 12 branches, the total probability of 12 leads is 12 × 8 = 96, plus DFs (we use seven HRV features, age, and sex features), then we finally get 105-dimensional features to construct a feature vector as input to the XGBoost model. The architecture of the CBi-DF-XGBoost fusion model is shown in Figure 1.

### 2.4 Training and Classification

To fully utilize the entire training set, a 5-fold cross-validation strategy was employed in this work. The original training set was randomly divided into five subsets. Each of the five subsets took turns as the validation set, and the remaining subsets were used as the training set. This approach was iterated five times by shifting the test data. The performances were evaluated in each iteration. Finally, the performances recorded in all five iterations were averaged and considered as the overall performance of our proposed system. Our model is also evaluated on the test set.

The experiments were performed on a computer with one CPU at 2.6 GHz, one NVIDIA GeForce RTX2060 GPU and 64-Gb memory. All the proposed models are run over a highly efficient GPU using the Keras deep learning framework (Chollet, 2018). We use stochastic gradient descent (SGD) combined with the momentum optimization algorithm to train the CNN-BiLSTM model. The SGD + Momentum algorithm more easily finds a flatter minimum than the Adam algorithm. Then, XGBoost conducts fusion training on the CNN-BiLSTM network array and the domain-specific features. For ablation studies, we also experimented with 12 independent CNN-BiLSTMs and the fusion model (CBi-XGBoost) that only fused 12 single-lead models by the XGBoost (Ye and Lu, 2020). The selection of hyperparameters for the models is shown in Table 2.

**TABLE 2:** Hyperparameters of the models.

Hyperparameter	Value	
	CBi-DF-XGBoost	CBi-XGBoost
**Boost**	gbtree	gbtree
**Learning Rate**	0.1	0.1
**Max_depth**	5	10
**Min_child_weight**	4	2.6
**Colsample_bytree**	0.8	0.7
**Colsample_bynode**	0.2	1
**Subsample**	0.6	0.7
**Max_delta_step**	0	5
**Eval_metric**	merror	merror
**Gamma**	0.4	0.3
**Num_round**	108	120

## 3 Results

### 3.1 Evaluation Metrics

In this paper, the average precision, accuracy, recall rate, F1 score, receiver operating characteristic (ROC) curve and area under the curve (AUC) value are adopted to measure the classification performance. The details are as follows:

$$Precision=\frac{TP}{TP+FP}\;$$ (3)

$$Accuracy=\frac{TP+TN}{Total}\;$$ (4)

$$Recall=\frac{TP}{TP+FN}\;$$ (5)

$$F1=\frac{2\left(Precision\times Recall\right)}{Precision+Recall}\;$$ (6)

For a certain class in the multiclassification problem, TP is the true positive, which indicates the number of correctly classified samples in this class, TN is the true negative, which indicates the number of samples that do not belong to this class and are not correctly classified into this class. FN is the false negative, which refers to the number of samples belonging to this class that are misclassified into other classes and FP denotes the false-positive positive, which indicates the number of samples misclassified in this class (Aziz et al., 2019). In our experiments, we use the average value among classes to evaluate the final performance of the fusion model. The F1 score is a comprehensive evaluation index that measures precision and recall (Flach and Kull, 2015). Among these metrics, the F1 score mainly assesses the recognition effect, which is the most important evaluation metric in the dataset. To analyse the performance of the model more intuitively, the confusion matrix is used to evaluate the prediction results.

### 3.2 Performance Evaluation

**FIGURE 6:**
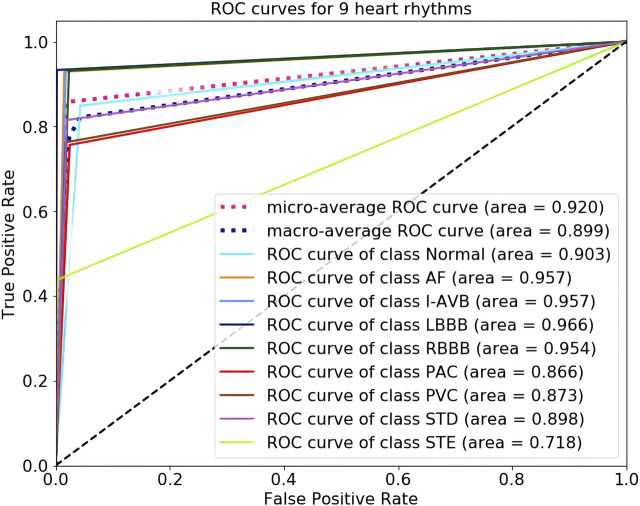
ROC curves for nine heart-rhythm predictions.

The 5-fold cross-validation diagnostic performance of CBi-DF-XGBoost is presented in Table 3. CBi-DF-XGBoost achieved a mean accuracy of 0.968 for the classification of the nine heart rhythms. The mean precision and recall were 0.857 and 0.814, respectively. The macro-average F1 score, which represented the harmonic mean of precision and recall was 0.825 for CBi-DF-XGBoost. The micro-average AUC value and macro-average AUC value were 0.919 and 0.898, respectively. Moreover, Figure 6 shows the ROC curve of the median macro-average AUC value for 5-fold cross-validation.

**TABLE 3:** 5-fold cross-validation diagnostic performance of CBi-DF-XGBoost.

Type	Accuracy	AUC	F1 score	Precision	Recall
**Normal**	0.941	0.903	0.801	0.758	0.849
**AF**	0.975	0.957	0.929	0.929	0.929
**I-AVB**	0.977	0.957	0.889	0.849	0.932
**LBBB**	0.996	0.966	0.933	0.933	0.933
**RBBB**	0.957	0.954	0.926	0.920	0.932
**PAC**	0.960	0.866	0.747	0.737	0.757
**PVC**	0.961	0.873	0.797	0.835	0.763
**STD**	0.961	0.898	0.828	0.842	0.815
**STE**	0.978	0.708	0.571	0.909	0.417
**Macro-average**	0.968	0.898	0.825	0.857	0.814

As shown in Table 3 and Figure 6, we noticed that the F1 scores of five heart rhythm types (AF, I-AVB, LBBB, RBBB, and STD) were higher than those of the normal type, reflecting the model’s ability to effectively identify abnormal ECG signals, with the normal type being one of the more difficult-to-predict types. Indeed, almost all of the top models produced very high F1 scores (>0.9) for AF and bundle branch blocks. The prediction of CBi-DF-XGBoost had the highest F1 score (0.933) and AUC value (0.966) on LBBB and a poor performance on PAC. The prediction of CBi-DF-XGBoost had the lowest F1 score (0.571) and AUC value (0.708) on STE, which may be due in part to physicians’ various opinions on how to diagnose STE (McCabe et al., 2013).

### 3.3 Classification Performance Comparison

To evaluate the performance of the proposed model, we choose some common network structures and state-of-art ECG classification algorithms to conduct experiments under the similar conditions and then compare the classification performance on the test set. Three common deep neural network frameworks, VGG (Simonyan and Zisserman, 2015), ResNet (He et al., 2016), and LSTM (Hochreiter and Schmidhuber, 1997), are adopted for performance comparison. Both the VGG and the ResNet networks are classical CNNs for processing images and signals. VGG based on 1-D convolution has been widely used in many signal processing tasks. In our experiments, we choose the VGG net with 16 1-D convolutional layers for comparison. ResNet designs a residual learning framework using shortcut identity connections to ease the training of very deep networks and make feature maps from shallower layers available at later stages. In our experiments, we use the 20-layer 1-D ResNet for comparison. Furthermore, two state-of-the-art ECG analysis methods are also used for performance comparison. Acharya et al. (Acharya et al., 2017) implemented an 11-layer CNN algorithm for the automated detection of normal and myocardial infarction ECG signals. (Fan et al., 2018) proposed a multiscale CNN (MSCNN) for screening AF recordings from ECG records. Both methods have achieved excellent classification results at present for the ECG classification task. In addition, we also compare the proposed method with some of the latest reported algorithms [(He et al., 2019) and (Yao et al., 2020) methodsGHz] that use the same dataset that we use. (Yao et al., 2020) improved the DNN used in their previous work (Yao et al., 2018), introducing an attention module after CNN and LSTM layers. This work gave greater weights to features extracted from more informative signal segments.

Table 4 shows the class-level F1 score and the average F1 score of eight reference models and our method. As can be observed, the proposed CBi-DF-XGBoost performs favourably against other models in terms of the F1 score. Specifically, compared with the plain VGG and ResNet networks, approximately 4.7% (0.828–0.781) and 5.5% (0.828–0.773) improvements are obtained by the proposed approach for the average F1 score, respectively. Our method obtains a 6.8% (0.828–0.760) gain compared to LSTM. Acharya et al. and Fan et al. are surpassed by our method in average F1 scores by 6.4% (0.828–0.764) and 3.2% (0.828–0.796), respectively. Compared with He et al. And Yao et al., our method increased the average F1 score by approximately 2.2% (0.828–0.806) and 1.6% (0.828–0.812), respectively. Furthermore, for each individual class of N, AF, I-AVB, LBBB, RBBB, PAC, PVC, STD, and STE, the gains in the F1 score were almost the highest. In particular, for a single disease, the F1 score increased by 12.0 and 14.0% in detecting paroxysmal arrhythmias (PACs) compared with ResNet and LSTM. Our method is 13.0 and 11.0% higher than the methods proposed by Acharya et al. and ResNet in detecting ST-segment elevated, respectively. Therefore, the comparison shown in Table 4 illustrates the effectiveness of our model.

**TABLE 4:** Classification performance on the dataset.

Type	F1 score							
	VGG	ResNet	LSTM	Acharya et al	Fan et al	He et al	Yao et al.	Our model
**Normal**	0.77	0.75	0.73	0.70	0.78	—	0.79	**0.81**
**AF**	0.87	0.90	0.92	0.90	0.92	—	0.92	**0.93**
**I-AVB**	0.79	0.84	0.77	0.75	0.80	—	0.85	**0.89**
**LBBB**	0.88	0.86	0.87	0.83	0.88	—	0.87	**0.93**
**RBBB**	0.90	0.91	0.92	0.92	0.92	—	**0.93**	**0.93**
**PAC**	0.68	0.63	0.61	0.70	**0.76**	—	0.74	0.75
**PVC**	0.82	0.82	0.80	0.85	0.83	—	**0.86**	0.79
**STD**	0.80	0.77	0.70	0.75	0.76	—	0.79	**0.83**
**STE**	0.52	0.49	0.51	0.47	0.51	—	0.56	**0.60**
**Average F1 Score**	0.781	0.773	0.760	0.764	0.796	0.806	0.812	**0.828**

The mark “—” is filled when the data was not given.

The bold values are the highest F1 score per line.

### 3.4 Ablation Studies

To analyse the relative contributions of the different components of our CBi-DF-XGBoost model, we evaluate some variants of the proposed method with different settings, including twelve independent single-lead models (CNN-BiLSTMs) and a fusion model (CBi-XGBoost) that only fused twelve CNN-BiLSTMs by the XGBoost.

#### 3.4.1 Single-Lead Models vs. 12-Lead Fusion Model

As shown in Table 5, we compare the F1 scores of classification prediction using CNN-BiLSTMs and CBi-XGBoost. In this experiment, we performed cross-validation experiments on the classification task for 12 leads. The CBi-XGBoost fused twelve CNN-BiLSTMs by XGBoost for the classification task. By comparison, we notice that CBi-XGBoost has a better performance than CNN-BiLSTMs. The best and worst F1 scores of CNN-BiLSTMs are aVR lead CNN-BiLSTM (0.700) and aVL lead CNN-BiLSTM (0.571), respectively. The F1 score of CBi-XGBoost is 0.806, which is an increase of 10.6% (0.806–0.700) to 23.5% (0.806–0.571) compared with CNN-BiLSTMs. The above comparison verifies that for S12L-ECGs, the model can obtain a better classification performance by fusing features of all leads.

**TABLE 5:** Classification performance comparison between single-lead models and CBi-XGBoost.

Model	F1 Score
I lead CNN-BiLSTM	0.680
II lead CNN-BiLSTM	0.680
III lead CNN-BiLSTM	0.601
aVR lead CNN-BiLSTM	0.700
aVL lead CNN-BiLSTM	0.571
aVF lead CNN-BiLSTM	0.663
V1 lead CNN-BiLSTM	0.665
V2 lead CNN-BiLSTM	0.652
V3 lead CNN-BiLSTM	0.699
V4 leadCNN-BiLSTM	0.688
V5 lead CNN-BiLSTM	0.699
V6 lead CNN-BiLSTM	0.658
CBi-XGBoost	**0.806**

The bold value is the highest F1 score of the models.

#### 3.4.2 Fusion Without Domain-Specific Features vs. Fusion With Domain-Specific Features

**FIGURE 7:**
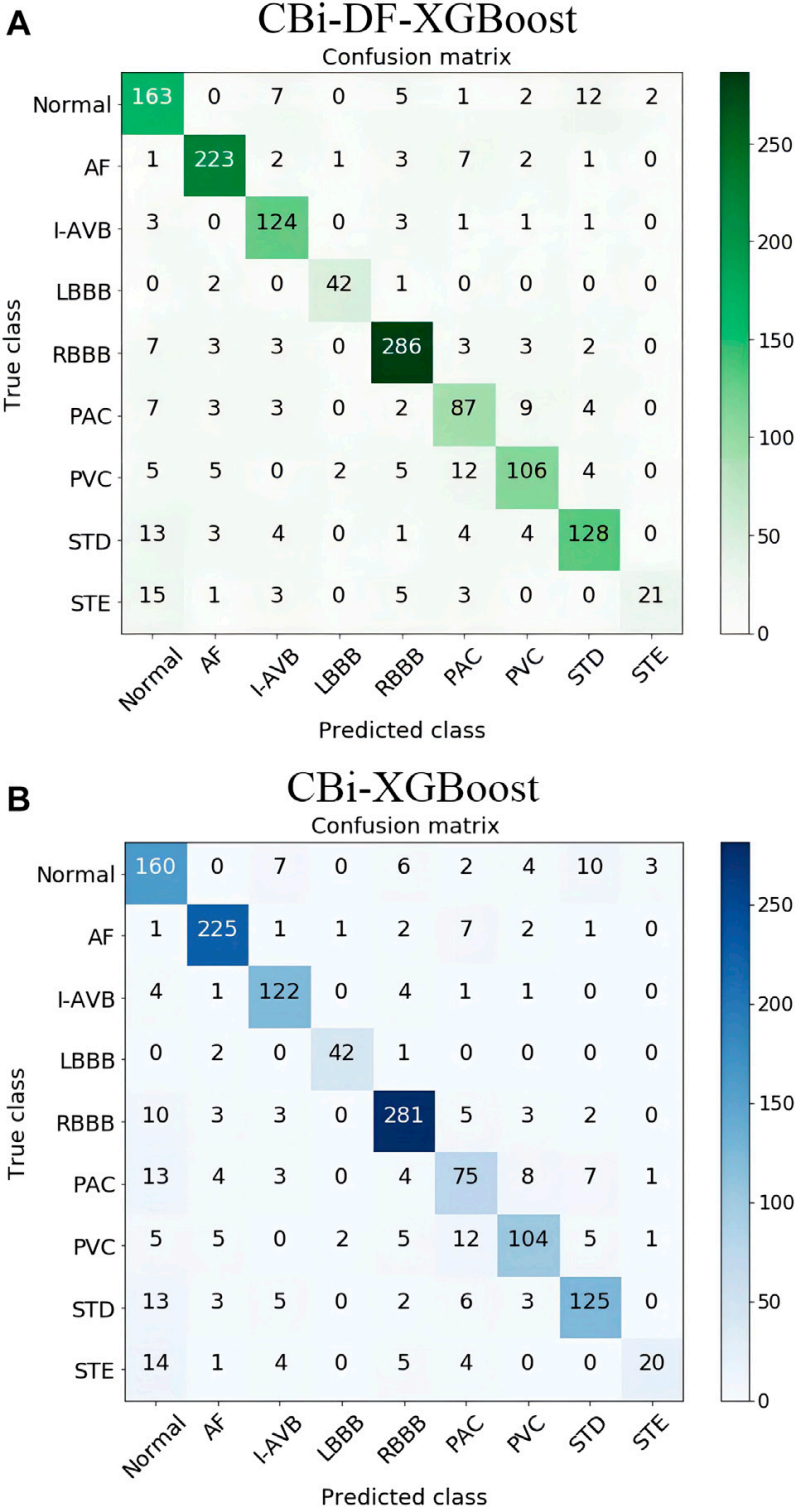
**(A)** Confusion matrix of CBi-DF-XGBoost at its median F1 score for 5-fold cross-validation **(B)** Confusion matrix of CBi-XGBoost at its median F1 score for 5-fold cross-validation. The ordinate is the nine label categories of the data, and the abscissa is the predicted category output by the model. The number of elements on the diagonal of the matrix is the number of correct classifications, and the remaining squares show the number of incorrect classifications.

To investigate the effect of the exclusion of DFs on this classification problem and the fusion model, we compared CBi-DF-XGBoost with the model without DFs (CBi-XGBoost). In the experiment, except for the CBi-DF-XGBoost model fusion with DFs and the CBi-XGBoost model fusion without DFs, the fusion models were subjected to a 5-fold cross-validation under the similar conditions to obtain the average F1 score values for comparison. The experimental results are shown in Table 6. Compared to CBi-XGBoost, the macro-average precision and recall rate of CBi-DF-XGBoost increased by 2.8 and 1.7%, respectively, the macro-average F1 score increased by approximately 1.9%, and the macro-average AUC value increased by 1.0%. Figure 7 shows the confusion matrix of the two models at their median F1 scores for 5-fold cross-validation. From the figures, it is clear that CBi-DF-XGBoost better distinguishes between normal signals and abnormal signals, especially PAC. This shows that the auxiliary information is helpful for improving the classification performance of the fusion model.

**TABLE 6:** Classification performance comparison between CBi-XGBoost and CBi-DF-XGBoost.

Model	AUC	F1 score	Precision	Recall
**CBi-XGBoost**	0.888	0.806	0.829	0.797
**CBi-DF-XGBoost**	0.898	0.825	0.857	0.814

## 4 Discussions

Our proposed fusion model (CBi-DF-XGBoost) can effectively fuse 12 single-lead CNN-BiLSTM models and domain-specific features, and achieve excellent performance. However, the following problems still exist for further study.

### 4.1 The Problem of Inter-subject Variations

Inter-subject variations in the ECG signals will affect the performance of the model when applied to new patients. In this paper, we are motivated to address this problem by adding age and sex features to the model to characterize the inter-subject variations in the ECG signals. Through experimental comparison, the performance of the model has been improved to some extent. However, it may not be enough to consider only these two features. As Gyawali et al. proved in their continuous researches (Gyawali et al., 2020; Gyawali et al., 2022), disentangling factors of anatomical variations from the ECG data can benefit the downstream task confounded by such anatomical variations. Their work suggests the important research direction to deal with the presence of significant inter-subject variations during an automated analysis of ECG data. In the future, utilizing the studies of inter-subject variations such as that presented in (Gyawali et al., 2020; Gyawali et al., 2022) may better solve the problem of inter-subject variations, so as to more accurately classify ECG signals automatically.

### 4.2 The Problem of Records With Multiple Labels

Records with multiple labels exist in part of the dataset because the patient providing the ECG record had more than one disease at the same time. For records with multiple labels, we involved them in training and testing as other records. We use the first label for training. In testing, for such records, we consider this to be a correct result as long as the classification result is consistent with one of the labels. However, This approach has two limitations. First, the features of other diseases in the records may interfere with the learning of the first-labeled features during training, thus reducing the system performance. Second, only one disease can be detected for each patient. And it is better to detect all the diseases that appear in the same record. How to improve the fusion model proposed in this paper to overcome these two limitations is one of our next research directions.

### 4.3 The Architecture of Single-Lead Models

Since our study focused on the overall benefits of model and domain-specific features fusion, we did not focus more on how to design better single-lead models. However, better single-lead models should benefit the overall performance of the fusion model. Future studies could investigate how different S12L-ECG types might be better modelled by using information from different single leads, whether different methods or different network architectures should be used for different single leads. In the future, we will continue to try other structures in the deep learning network to further improve the performance of the fusion model.

### 4.4 Different Leads Contribute Differently to Arrhythmia Detection

We looked at the classification performance of each single-lead model on each specific diagnosis. We found that some of the single-lead models performed well on a specific diagnosis (such as the models for leads I and V1 excelled on the diagnosis of LBBB. The F1 score of the I lead CNN-BiLSTM on LBBB was 0.934, while its mean F1 score on all classifications was only 0.680. The F1 score of the V1 lead CNN-BiLSTM on LBBB was 0.933, while its mean F1 score on all classifications was only 0.665), which is also consistent with the studies in (Dale, 2000; Surawicz et al., 2009; Ikeda, 2021) that LBBB is diagnosed by distinct QRS morphology at leads I, aVL, V1, V2, V5, and V6. However, we need more research to obtain definitive statistical results, including a comprehensive consideration of the interference of the records with multiple labels, the suitability of single-lead models for specific diagnoses, and other issues. With a view to further investigate the different contributions of different leads to arrhythmia detection. To investigate how to perform multi-lead model fusion more effectively.

## 5 Conclusion

In this paper, we present a novel fusion model (CBi-DF-XGBoost) for S12L-ECG record classification by using XGBoost to fuse 12 single-lead CNN-BiLSTM models and DFs. Ablation studies verify that the complementary features from the different channels of the S12L-ECG signal and the auxiliary information of DFs are helpful to improve the performance of the fusion model. Furthermore, we demonstrate an outstanding performance for S12L-ECG classification on the dataset compared with some existing methods. The results of the comparative experiments confirm the effectiveness of the proposed fusion method. We will apply CBi-DF-XGBoost to other physiological signal analyses and processing requirements.

## Data Availability

The original contributions presented in the study are included in the article/Supplementary Material, further inquiries can be directed to the corresponding author.

## References

[B1] Acharya U. R. Fujita H. Oh S. L. Hagiwara Y. Tan J. H. Adam M. (2017). Application of Deep Convolutional Neural Network for Automated Detection of Myocardial Infarction Using ECG Signals. Inf. Sci. 415-416, 190–198. 10.1016/j.ins.2017.06.027

[B2] Afonso V. X. Tompkins W. J. (1995). Detecting Ventricular Fibrillation. IEEE Eng. Med. Biol. Mag. 14, 152–159. 10.1109/51.376752

[B3] Alcaraz R. Abásolo D. Hornero R. Rieta J. J. (2010). Optimal Parameters Study for Sample Entropy-Based Atrial Fibrillation Organization Analysis. Comput. Methods Programs Biomed. 99, 124–132. 10.1016/j.cmpb.2010.02.009 20392514

[B4] Alyasseri Z. A. A. Khader A. T. Al-Betar M. A. Awadallah M. A. (2018). Hybridizing β-hill Climbing with Wavelet Transform for Denoising ECG Signals. Inf. Sci. 429, 229–246. 10.1016/j.ins.2017.11.026A

[B5] Anderson M. E. Kass R. S. Clancy C. E. (2010). Basis and Treatment of Cardiac Arrhythmias. Germany: Springer.

[B6] Ashley E. A. Niebauer J. (2004). Cardiology Explained. London: Andrew Ward. 20821845

[B7] Aziz S. R. Khan T. A. Nadeem A. (2019). Experimental Validation of Inheritance Metrics' Impact on Software Fault Prediction. IEEE Access 7, 85262–85275. 10.1109/ACCESS.2019.2924040

[B8] Baloglu U. B. Talo M. Yildirim O. Tan R. S. Acharya U. R. (2019). Classification of Myocardial Infarction with Multi-lead ECG Signals and Deep CNN. Pattern Recognition Lett. 122, 23–30. 10.1016/j.patrec.2019.02.016

[B9] Banerjee S. Mitra M. (2014). Application of Cross Wavelet Transform for ECG Pattern Analysis and Classification. IEEE Trans. Instrum. Meas. 63, 326–333. 10.1109/TIM.2013.2279001

[B10] Bin Y. Yang Y. Shen F. Xie N. Shen H. T. Li X. (2019). Describing Video with Attention-Based Bidirectional LSTM. IEEE Trans. Cybern. 49, 2631–2641. 10.1109/TCYB.2018.2831447 29993730

[B11] Chen T. Guestrin C. (2016). “XGBoost,” in 22nd ACM SIGKDD International Conference ACM, New York, USA. 10.1145/2939672.2939785

[B12] Chollet F. (2018). Keras: The Python Deep Learning Library. Available at: https://keras.io.

[B13] Dale D. (2000). Cardiology - Rapid Interpretation of EKG’s. Tampa, Fla: Cover publishing company.

[B14] Dilmac S. Korurek M. (2015). ECG Heart Beat Classification Method Based on Modified ABC Algorithm. Appl. Soft Comput. 36, 641–655. 10.1016/j.asoc.2015.07.010

[B15] Fan X. Yao Q. Cai Y. Miao F. Sun F. Li Y. (2018). Multiscaled Fusion of Deep Convolutional Neural Networks for Screening Atrial Fibrillation from Single Lead Short ECG Recordings. IEEE J. Biomed. Health Inform. 22, 1744–1753. 10.1109/JBHI.2018.2858789 30106699

[B16] Flach P. A. Kull M. (2015). “Precision-Recall-Gain Curves: PR Analysis Done Right,” in Proceedings of the 28th International Conference on Neural Information Processing Systems, Montreal, QC, Canada, December 7–12, 2015, 838–846.

[B17] Fred K. (2020). ECG Interpretation: From Pathophysiology to Clinical Application. Germany: Springer.

[B18] Fukushima K. Miyake S. (1982). Neocognitron: A Self-Organizing Neural Network Model for a Mechanism of Visual Pattern Recognition. IEEE Trans. Syst. Man Cybernetics 45, 267–285. 10.1007/978-3-642-46466-9_18

[B19] Goller C. Kuchler A. (1996). “Learning Task-dependent Distributed Representations by Backpropagation through Structure,” in Proceedings of International Conference on Neural Networks, Washington, DC, USA, 347–352. 10.1109/ICNN.1996.548916

[B20] Graves A. Mohamed A.-R. Hinton G. (2013). “Speech Recognition with Deep Recurrent Neural Networks,” in IEEE International Conference on Acoustics, Speech and Signal Processing, Vancouver, BC, Canada, 6645–6649. 10.1109/ICASSP.2013.6638947

[B21] Gyawali P. K. Horacek B. M. Sapp J. L. Wang L. (2020). Sequential Factorized Autoencoder for Localizing the Origin of Ventricular Activation from 12-lead Electrocardiograms. IEEE Trans. Biomed. Eng. 67 (5), 1505–1516. 10.1109/TBME.2019.2939138 31494539 PMC7051887

[B22] Gyawali P. K. Murkute J. V. Toloubidokhti M. Jiang X. Horacek B. M. Sapp J. L. (2022). Learning to Disentangle Inter-subject Anatomical Variations in Electrocardiographic Data. IEEE Trans. Biomed. Eng. 69 (2), 860–870. 10.1109/TBME.2021.3108164 34460360 PMC8858595

[B23] Hannun A. Y. Rajpurkar P. Haghpanahi M. Tison G. H. Bourn C. Turakhia M. P. (2019). Cardiologist-level Arrhythmia Detection and Classification in Ambulatory Electrocardiograms Using a Deep Neural Network. Nat. Med. 25, 65–69. 10.1038/s41591-018-0268-3 30617320 PMC6784839

[B24] He K. Zhang X. Ren S. Sun J. (2016). “Deep Residual Learning for Image Recognition,” in IEEE Conference on Computer Vision and Pattern Recognition (CVPR), Las Vegas, NV, United states, June 26–July 1, 2016, 770–778. 10.1109/CVPR.2016.90

[B25] He R. Liu Y. Wang K. Zhao N. Yuan Y. Li Q. (2019). Automatic Cardiac Arrhythmia Classification Using Combination of Deep Residual Network and Bidirectional LSTM. IEEE Access 7, 102119–102135. 10.1109/ACCESS.2019.2931500

[B26] Hochreiter S. Schmidhuber J. (1997). Long Short-Term Memory. Neural Comput. 9, 1735–1780. 10.1162/neco.1997.9.8.1735 9377276

[B27] Ikeda T. (2021). Right Bundle Branch Block: Current Considerations. Ccr 17 (1), 24–30. 10.2174/1573403X16666200708111553 PMC8142372 32640959

[B28] Ioffe S. Szegedy C. (2015). Batch Normalization: Accelerating Deep Network Training by Reducing Internal Covariate Shift. ArXiv.

[B29] Jordaens L. (2018). A Clinical Approach to Arrhythmias Revisited in 2018. Neth. Heart J. 26, 182–189. 10.1007/s12471-018-1089-1 29450695 PMC5876171

[B30] Joshi A. K. Tomar A. Tomar M. (2014). A Review Paper on Analysis of Electrocardiograph (ECG) Signal for the Detection of Arrhythmia Abnormalities. Ijareeie 03, 12466–12475. 10.15662/ijareeie.2014.0310028

[B31] Kamphaus R. W. (2000). Clinical Assessment of Child and Adolescent Intelligence. Germany: Springer.

[B32] Ker J. Bai Y. Lee H. Y. Rao J. P. Wang L. (2019a). Automated Brain Histology Classification Using Machine Learning. J. Clin. Neurosci. 66, 239–245. 10.1016/j.jocn.2019.05.019 31155342

[B33] Ker J. Singh S. P. Bai Y. Rao J. Lim T. Wang L. (2019b). Image Thresholding Improves 3-Dimensional Convolutional Neural Network Diagnosis of Different Acute Brain Hemorrhages on Computed Tomography Scans. Sensors 19, 2167. 10.3390/s19092167 PMC6539746 31083289

[B34] Ker J. Wang L. Rao J. Lim T. (2018). Deep Learning Applications in Medical Image Analysis. IEEE Access 6, 9375–9389. 10.1109/ACCESS.2017.2788044

[B35] Lacalzada‐Almeida J. Izquierdo‐Gómez M. M. Laynez‐Cerdeña I. García‐Niebla J. Bruña V. Bayés de Luna A. (2020). Electrocardiogram and Left Atrial Abnormality: Design of an Observational Study to Clarify Diagnostic Criteria. Ann. Noninvasive Electrocardiol. 25 (6). 10.1111/anec.12770 PMC7679830 32468671

[B36] Li K. Pan W. Li Y. Jiang Q. Liu G. (2018). A Method to Detect Sleep Apnea Based on Deep Neural Network and Hidden Markov Model Using Single-lead ECG Signal. Neurocomputing 294, 94–101. 10.1016/j.neucom.2018.03.011

[B37] Li Z. Zhou D. Wan L. Li J. Mou W. (2020). Heartbeat Classification Using Deep Residual Convolutional Neural Network from 2-lead Electrocardiogram. J. Electrocardiol. 58, 105–112. 10.1016/j.jelectrocard.2019.11.046 31812617

[B38] Lin C.-H. (2008). Frequency-domain Features for ECG Beat Discrimination Using Grey Relational Analysis-Based Classifier. Comput. Maths. Appl. 55, 680–690. 10.1016/j.camwa.2007.04.035

[B39] Liu F. Liu C. Zhao L. Zhang X. Wu X. Xu X. (2018). An Open Access Database for Evaluating the Algorithms of Electrocardiogram Rhythm and Morphology Abnormality Detection. J Med. Imaging Hlth Inform. 8, 1368–1373. 10.1166/jmihi.2018.2442

[B40] Liu F. Zhou X. Cao J. Wang Z. Wang H. Zhang Y. (2019). A LSTM and CNN Based Assemble Neural Network Framework for Arrhythmias Classification. IEEE Int. Conf. Acoust. Speech Signal Process. 2019, 1303–1307. 10.1109/ICASSP.2019.8682299

[B41] Malik M. Bigger J. T. Camm A. J. Kleiger R. E. Malliani A. Moss A. J. (1996). Heart Rate Variability: Standards of Measurement, Physiological Interpretation, and Clinical Use. Eur. Heart J. 17, 354–381. 10.1111/j.1542-474X.1996.tb00275.x 8737210

[B42] Martis R. J. Acharya U. R. Min L. C. (2013). ECG Beat Classification Using PCA, LDA, ICA and Discrete Wavelet Transform. Biomed. Signal Process. Control. 8, 437–448. 10.1016/j.bspc.2013.01.005

[B43] Mateo J. Torres A. M. Aparicio A. Santos J. L. (2016). An Efficient Method for ECG Beat Classification and Correction of Ectopic Beats. Comput. Electr. Eng. 53, 219–229. 10.1016/j.compeleceng.2015.12.015

[B44] Mccabe J. M. Armstrong E. J. Ku I. Kulkarni A. Hoffmayer K. S. Bhave P. D. (2013). Physician Accuracy in Interpreting Potential ST‐Segment Elevation Myocardial Infarction Electrocardiograms. Jaha 2, 268. 10.1161/JAHA.113.000268 PMC3835230 24096575

[B45] Mikolov T. Karafiát M. Burget L. Cernocký J. H. Khudanpur S. (2010). “Recurrent Neural Network Based Language Model,” in 11th Annual Conference of the International Speech Communication Association, Makuhari, Chiba, Japan, September 26-30, 2010.

[B46] Minami K. Nakajima H. Toyoshima T. (1999). Real-time Discrimination of Ventricular Tachyarrhythmia with Fourier-Transform Neural Network. IEEE Trans. Biomed. Eng. 46, 179–185. 10.1109/10.740880 9932339

[B47] Ren Y. Teng C. Li F. Chen B. Ji D. (2017). Relation Classification via Sequence Features and Bi-directional LSTMs. Wuhan Univ. J. Nat. Sci. 22, 489–497. 10.1007/s11859-017-1278-6

[B48] Roth G. A. Abate D. Abate K. H. Abay S. M. Abbafati C. Abbasi N. (2018). Global, Regional, and National Age-sex-specific Mortality for 282 Causes of Death in 195 Countries and Territories, 1980-2017: a Systematic Analysis for the Global Burden of Disease Study 2017. Lancet 392, 1736–1788. 10.1016/S0140-6736(16)31460-X10.1016/S0140-6736(18)32203-7 30496103 PMC6227606

[B49] Saxena S. Vijay R. (2020). Optimal Selection of Wavelet Transform for De-noising of ECG Signal on the Basis of Statistical Parameters. Adv. Intell. Syst. Comput. 1118, 731–739. 10.1007/978-981-15-2475-2_67

[B50] Schläpfer J. Wellens H. J. (2017). Computer-Interpreted Electrocardiograms. J. Am. Coll. Cardiol. 70, 1183–1192. 10.1016/j.jacc.2017.07.723 28838369

[B51] Shadmand S. Mashoufi B. (2016). A New Personalized ECG Signal Classification Algorithm Using Block-Based Neural Network and Particle Swarm Optimization. Biomed. Signal Process. Control. 25, 12–23. 10.1016/j.bspc.2015.10.008

[B52] Shaker A. M. Tantawi M. Shedeed H. A. Tolba M. F. (2020). Generalization of Convolutional Neural Networks for ECG Classification Using Generative Adversarial Networks. IEEE Access 8, 35592–35605. 10.1109/ACCESS.2020.2974712

[B53] Shih J.-Y. Chen Z.-C. Chang H.-Y. Liu Y.-W. Ho C.-H. Chang W.-T. (2019). Risks of Age and Sex on Clinical Outcomes post Myocardial Infarction. IJC Heart & Vasculature 23, 100350. 10.1016/j.ijcha.2019.100350 30976655 PMC6441739

[B54] Simonyan K. Zisserman A. (2015). Very Deep Convolutional Networks for Large-Scale Image Recognition.

[B55] Surawicz B. Childers R. W. Deal B. Gettes L. S. Bailey J. J. Gorgels A. P. M. (2009). AHA/ACCF/HRS Recommendations for the Standardization and Interpretation of the Electrocardiogram: Part III: Intraventricular Conduction Disturbances: a Scientific Statement from the American Heart Association Electrocardiography and Arrhythmias Committee, Council on Clinical Cardiology; the American. J. Am. Coll. Cardiol. 53, 976–981. 10.1016/j.jacc.2008.12.013 19281930

[B56] Tateno K. Glass L. (2001). Automatic Detection of Atrial Fibrillation Using the Coefficient of Variation and Density Histograms of RR and ΔRR Intervals. Med. Biol. Eng. Comput. 39, 664–671. 10.1007/BF02345439 11804173

[B57] Tseng K.-K. Lee D. Chen C. B. (2016). ECG Identification System Using Neural Network with Global and Local Features. Melbourne: International Association for Development of the Information Society.

[B58] Uhlig S. Meylan A. Rudolph U. (2020). Reliability of Short-Term Measurements of Heart Rate Variability: Findings from a Longitudinal Study. Biol. Psychol. 154, 107905. 10.1016/j.biopsycho.2020.107905 32505705

[B59] Wang C. Guo J. (2019). A Data-Driven Framework for Learners' Cognitive Load Detection Using ECG-PPG Physiological Feature Fusion and XGBoost Classification. Proced. Comput. Sci. 147, 338–348. 10.1016/j.procs.2019.01.234

[B60] Wilson F. N. Kossmann C. E. Burch G. E. Goldberger E. Graybiel A. Hecht H. H. (1954). Recommendations for Standardization of Electrocardiographic and Vectorcardiographic Leads. Circulation 10, 564–573. 10.1161/01.CIR.10.4.564 13209743

[B61] Yadav S. S. Jadhav S. M. (2019). Deep Convolutional Neural Network Based Medical Image Classification for Disease Diagnosis. J. Big Data 6, 113–131. 10.1186/s40537-019-0276-2

[B62] Yao Q. Fan X. Cai Y. Wang R. Yin L. Li Y. (2018). “Time-Incremental Convolutional Neural Network for Arrhythmia Detection in Varied-Length Electrocardiogram,” in IEEE 16th Intl Conf on Dependable, Autonomic and Secure Computing, 16th Intl Conf on Pervasive Intelligence and Computing, 4th Intl Conf on Big Data Intelligence and Computing and Cyber Science and Technology Congress, Athens, Greece, August 12–15, 2018, 754–761. 10.1109/DASC/PiCom/DataCom/CyberSciTec.2018.00131

[B63] Yao Q. Wang R. Fan X. Liu J. Li Y. (2020). Multi-class Arrhythmia Detection from 12-lead Varied-Length ECG Using Attention-Based Time-Incremental Convolutional Neural Network. Inf. Fusion 53, 174–182. 10.1016/j.inffus.2019.06.024

[B64] Ye X. Lu Q. (2020). “Automatic Classification of 12-lead ECG Based on Model Fusion,” in 13th International Congress on Image and Signal Processing, BioMedical Engineering and Informatics (CISP-BMEI), Chengdu, China, 733–738. 10.1109/CISP-BMEI51763.2020.9263559

[B65] Yildirim O. Baloglu U. B. Tan R.-S. Ciaccio E. J. Acharya U. R. (2019). A New Approach for Arrhythmia Classification Using Deep Coded Features and LSTM Networks. Comput. Methods Programs Biomed. 176, 121–133. 10.1016/j.cmpb.2019.05.004 31200900

